# Identifying hot spots for harm and blind spots across the care pathway from patient complaints about general practice

**DOI:** 10.1093/fampra/cmab109

**Published:** 2021-09-19

**Authors:** Emily O’Dowd, Sinéad Lydon, Kathryn Lambe, Chris Rudland, Aoife Hilton, Paul O’Connor

**Affiliations:** Discipline of General Practice, School of Medicine, National University of Ireland Galway, Galway, Ireland; Irish Centre for Applied Patient Safety and Simulation, National University of Ireland Galway, Galway, Ireland; Irish Centre for Applied Patient Safety and Simulation, National University of Ireland Galway, Galway, Ireland; School of Medicine, National University of Ireland Galway, Galway, Ireland; Health Research Board, Dublin, Ireland; National Complaints Governance and Learning Team, Health Service Executive, Limerick, Ireland; National Complaints Governance and Learning Team, Health Service Executive, Limerick, Ireland; Discipline of General Practice, School of Medicine, National University of Ireland Galway, Galway, Ireland; Irish Centre for Applied Patient Safety and Simulation, National University of Ireland Galway, Galway, Ireland

**Keywords:** doctor–patient relationship, patient complaints, patient safety, population health, primary care, quality of care

## Abstract

**Introduction:**

Healthcare complaints are underutilized for quality improvement in general practice. Systematic analysis of complaints has identified hot spots (areas across the care pathway where issues occur frequently) and blind spots (areas across the care pathway that cannot be observed by staff) in secondary care. The Healthcare Complaints Analysis Tool (HCAT) has been adapted to the HCAT(GP).

**Aims:**

This study aimed to: (i) assess whether the HCAT(GP) can systematically analyze complaints about general practice; and (ii) identify hot spots and blind spots in general practice.

**Methods:**

GP complaints were sampled. Complaints were coded with the HCAT(GP), classified by HCAT(GP) category (e.g. Safety, Environment, Listening), stage of care (e.g. accessing care, referral/follow-up), severity (e.g. low, medium, high), and harm (e.g. none, major). Descriptive statistics were run to identify discrete issues. A chi-square test of independence identified hot spots, and logistic regression was used for blind spots.

**Results:**

A total of 230 complaints, encompassing 432 issues (i.e. unique problems within complaints), were categorized. Relationship issues (e.g. problems with listening, communication, and patient rights) emerged most frequently (*n* = 174, 40%). Hot spots were identified in the consultation and the referral/follow-up stages (χ ^2^(5, *n* = 432) = 17.931, *P* < 0.05). A blind spot for multiple issues was identified, with the likelihood of harm increasing with number of issues (odds ratio = 2.02, confidence interval = 1.27–3.23, *P* < 0.05).

**Conclusions:**

Complaints are valuable data for improving general practice. This study demonstrated that the HCAT(GP) can support the systematic analysis of general practice complaints, and identify hot spots and blind spots in care.

**Key Messages**

- Complaints made about general practice are underutilized for quality improvement.
- The HCAT(GP) is a reliable tool for the collective analysis of GP complaints.
- Hot spots for harm emerged at the consultation stage of the care pathway.
- Blind spots in the care pathway emerged from the analysis.
- These findings have potential for supporting quality improvement.

## Introduction

Healthcare complaints are expressions of dissatisfaction with care provided.^1^ While complaints have typically been considered in terms of risk management, they can also be an opportunity to gain insight into patient perceptions of safety in healthcare.^2,3^ Patients are in a position to identify areas of risk within healthcare that are not discernible by staff.^4,5^ Therefore, incorporating patient insights into quality improvement can complement other measurement and monitoring tools.^3^ Healthcare complaints are increasingly considered as one means through which patient insights on care quality can be utilized.^1^ When using complaints for insight into quality and safety, the description of events within a complaint is taken at face value, without further scrutiny. Many complaints are not upheld following individual investigations,^6^ and therefore there is an argument that they can be considered an unreliable data source. However, whether or not the complaint is upheld in investigations is inconsequential,^3^ as the complainant saw a need to make a complaint, and that in itself is relevant to quality improvement. Healthcare complaints are therefore a burgeoning avenue for researchers looking to improve patient care.

When systematically analyzed, and considered through a quality improvement rather than a risk management lens,^7^ healthcare complaints have the potential for identifying hot spots (i.e. points in care with a high prevalence of harm or near-misses) and blind spots (i.e. points in care that cannot be observed by staff members).^2^ Hot spots for harm are areas across the care pathway where either harm or near-misses (issues which could lead to harm but did not), occur at high frequency.^2^ Blind spots are areas in healthcare where patients experience problems and staff do not have oversight, for example difficulties in accessing care.^2^ Examining hot spots and blind spots is a relatively new focus of quality improvement research, and have been explored in analyses of hospital complaints.^2^ Following the publication of the Francis report,^8^ using complaints to identify hot spots and blind spots has begun to receive more attention.^2^ The Healthcare Complaints Analysis Tool (General Practice), or HCAT(GP), provides a reliable approach to analyzing complaints about general practice.^9^ This tool was adapted directly from the HCAT for secondary care.^10^ HCAT(GP) has acceptable reliability when used to evaluate fictitious patient complaints.^9^ However, the use of the tool to analyze a database of real complaints about general practice has not been assessed. Therefore, the aims of this paper are to:

(1) assess whether the HCAT(GP) can be used to support the systematic analysis of actual patient complaints about general practice; and
(2) identify hot spots and blind spots in general practice.

## Methods

### Design

This study used a retrospective analysis of databases to sample and categorizes healthcare complaints about general practice in Ireland.

### Setting

There are a total of 29.1 million patient interactions with general practice in Ireland.^11^ One study of out-of-hours general practice recorded a complaints prevalence of 0.61 per 1,000 consultations,^12^ and another found that GPs are named in 47% of complaints.^13^ This high rate of complaints in general practice highlights the need to explore GP complaints for quality improvement.^7^ GP complaints in Ireland may be made either to the Health Service Executive (HSE), to the GP directly, or to the Irish Medical Council. There is no singular, comprehensive database of complaints.

### Sample

Two samples of complaints were analyzed for this study. The first sample (*n* = 69) was all of the 2019 general practice complaints received by the Irish Health Service Executive. These were collated by the National Complaints Governance and Learning Team from the HSE, by contacting complaints managers within local regions who forwarded the complaints to the central team until late 2019. The second sample of complaints (*n* = 161) was collated by an Irish medical indemnity company who represent a portion of Irish GPs, and constituted all complaints made to the Irish Medical Council from 2017 to 2019 (inclusive) about GPs insured with that company. These databases are likely to capture a mix of both public and private patients in the GP system, and reflect the 2 external complaints avenues available to patients. In order to ensure compliance with General Data Protection Regulations (GDPR), identifiable information was redacted by the data controllers prior to sharing with the research team.

### Ethical approval

This study received ethical approval from the NUI Galway Research Ethics committee (REC), reference number 18-Sept-17.

### HCAT(GP)

**Fig. 1.**
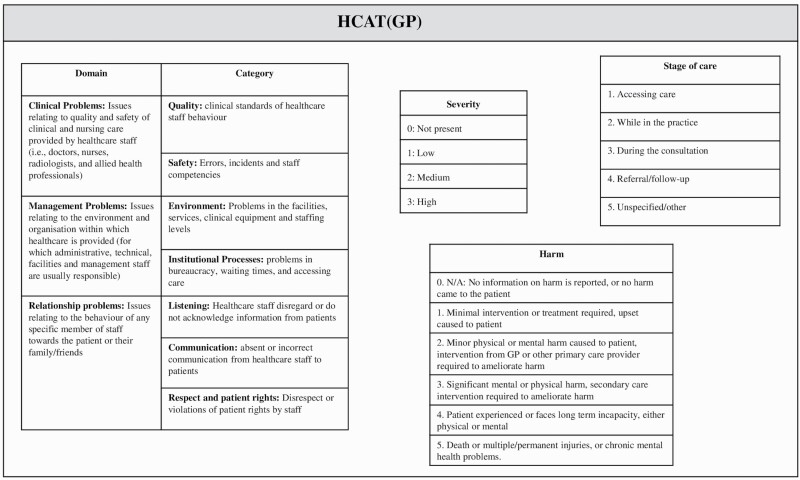
Healthcare Complaints Analysis Tool (General Practice) [HCAT(GP)].

All the complaints were analyzed using the HCAT(GP).^9^ The HCAT(GP) differs from the original HCAT for hospital complaints in 2 respects: (i) the stages of care and (ii) the examples provided for severity scales. Full information on the adaptation of the HCAT(GP) has been presented previously.^9^ It can be seen from Fig. 1 that HCAT(GP) supports the classification of issue(s) (i.e. unique problems mentioned by a complainant within a complaint) into HCAT(GP) categories, the stage of care at which the issue occurred, the severity of the issue(s) within the complaint, and the overall level of harm reported by the complainant.

### Procedure

The redacted complaints were collated in Excel. Each complaint was read by the primary researcher (EOD), and then the HCAT(GP) was used to categorize the complaint. Only one overall harm rating was captured for each complaint, as per the guidelines for the original HCAT,^10^ which require that only the highest level of harm within a complaint is reported.

In order to ascertain inter-rater reliability across the entire tool, a second researcher (KL) independently double-coded 33% of the sample using the HCAT(GP). It was decided to double code 33% of the sample based on previous research which used a minimum of 10%.^14,15^ Reliability for different elements of the HCAT(GP) has also been determined in a previous paper.^9^ Disagreements were resolved through discussion and consensus in order to allow for use of these complaints in the main analysis. Any missing demographic data were coded as “NA.” Data were cleaned and exported into R statistical software^16^ for analysis. The “Harm” variable was recoded into a binomial variable (i.e. a harm score of 0 on the HCAT(GP) was recoded to “No harm” (0), and scores from 1 to 5 were recoded as “Harm” (1)) This recoding, while losing some of the detail of the different levels of harm, was necessary to correctly run statistical tests due to the size of the sample. Analysis was then conducted on the cleaned data.

### Analysis

There were 3 stages to the analysis.

1. *Descriptive analysis*. Descriptive statistics were computed to determine frequent issues within the complaints. This was followed by the assessment of inter-rater reliability across all of the HCAT(GP) using Gwet’s AC1 (a measure of agreement between raters^17^).
2. *Identification of hot spots*. This analysis was based on existing R code.^2^ Hot spots were defined as points in care where harm or near-misses were prevalent. It was intended to analyze the near-miss hot spot in this study by examining the complaints which had high severity but no harm. However, this was not possible due to small sample size of the present study. We therefore only assessed for the harm hot spot. A chi-square test of independence including the variables of harm and stage of care was used to establish whether there was an association between the stage of care of an issue, and harm to the patient, and to identify at which stages in the care pathway harm was more or less likely to occur.
3. *Identification of blind spots*. Blind spots were defined as areas across the care pathway that are unobservable or difficult to observe. There are 3 main types of blind spot that can be captured by HCAT(GP). The first is the “entry/exit” blind spot, capturing issues that occur at the boundaries of care or outside of the general practice setting. The “errors of omission” blind spot, when an action is not done, is implicit within the HCAT(GP), as in the original HCAT.^2^ These were assessed using descriptive statistics. The final blind spot examined was the “systemic problems” blind spot; where issues occur across multiple stages of the care pathway. This was analyzed using a logistic regression with harm as the outcome variable, predicted by number of issues per complaint and number of stages of care within a complaint.

## Results

### Inter-rater reliability

Substantial inter-rater reliability across the entire HCAT(GP) was achieved between the 2 researchers (Gwet’s AC1 = 0.79, confidence interval [CI] = 0.77–0.82). Further detail on the reliability of the HCAT(GP) can be found in a previous paper.^9^

### Descriptive statistics

A total of 230 complaints about general practice were analyzed. Details of the descriptive statistics are shown in Table 1.

**Table 1.** Descriptive statistics of complaints (dating from 2017 to 2019).

Descriptive statistics	*N* (%) (total *n =* 230)
Data source	
Medical indemnity company	161 (70%)
HSE community healthcare organizations	69 (30%)
Complainant	
Patient	131 (57%)
Parent	34 (15%)
Child of patient	21 (9%)
Other family members	23 (10%)
Other	11 (5%)
No information	10 (4%)
Gender of staff member(s) complained against	
Female	52 (23%)
Male	101 (44%)
Female and male staff	11 (5%)
No information	66 (28%)

Table 2 provides an overview of the analysis. There were a total of 432 individual issues within the 230 complaints, with a mean of 1.88 problems per complaint (SD = 0.98). When analyzed, each of the 3 domains from the HCAT(GP) (i.e. Clinical, Management, and Relationship problems) were represented in the complaints. This reflects the patients coming into contact with both clinical and administrative aspects of healthcare in this context. Specific issues within the complaints occurred most frequently at the “Consultation” stage of care (*n* = 208, 48%). Half of the complaints reported some level of harm, and the majority of the issues were judged to have either medium (*n* = 178, 41%) or high (*n* = 165, 38%) severity (see Table 2).

**Table 2.** Complaints issues analyzed by HCAT(GP) (years 2017–2019).

HCAT(GP) sections	*N* (%) issues (total *n =* 432)	
Stages of care		
1. Accessing care	72 (17%)	
2. In the practice	25 (6%)	
3. During the consultation	208 (48%)	
4. Follow-up/referral	59 (14%)	
5. Other	45 (10%)	
Multiple stages	23 (5%)	
Severity		
1. Low	89 (21%)	
2. Medium	178 (41%)	
3. High	165 (38%)	
Domains Categories		Example issues within categories
Clinical domain	139 (32%)	
Quality	89 (21%)	Not conducting assessment of patient
Safety	50 (12%)	Misdiagnosis of appendicitis
Relationship domain	174 (40%)	
Listening	45 (10%)	Parent input on child illness ignored
Communication	27 (6%)	Blood test results not received by patient
Respect and patient rights	102 (24%)	Verbal assault of patient
Management domain	119 (28%)	
Environment	14 (3%)	Surgery not accessible by wheelchair user
Institutional processes	105 (24%)	Patient not able to register with GP
Harm	*N* (%) (total *n* = 230)	Example harm
0. No harm reported	115 (50%)	—
1. Minimal	57 (25%)	Complainant upset
2. Minor	22 (10%)	Patient experienced stress and anxiety
3. Moderate	14 (6%)	Short term recovery impacted
4. Major	6 (2%)	Patient developed post traumatic stress disorder
5. Catastrophic	16 (7%)	Patient died

### Hot spots

**Fig. 2.**
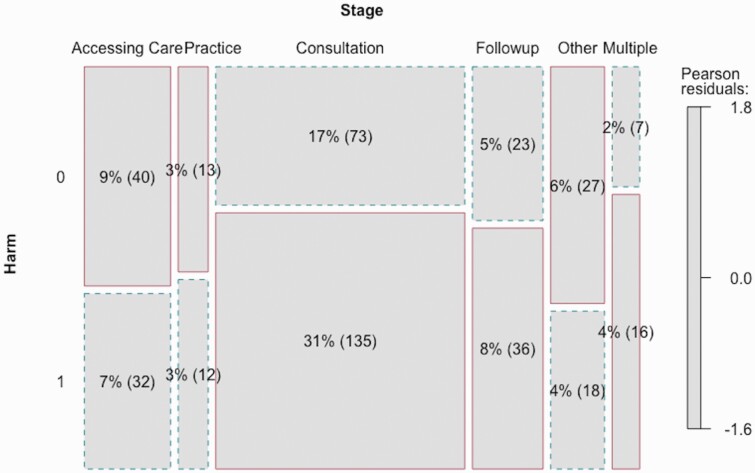
Matrix plot of harm by stages of care (Harm 0 = no harm present, Harm 1 = harm present).

Hot spots for harm were identified within the data. The chi-square test of independence between the harm and stage of care variables found a significant relationship between stage of care and whether or not harm was present in a complaint (χ ^2^(5, *n* = 432) = 17.931, *P* < 0.05). This indicated that certain stages of care, where harm frequently occurred could be considered “hot spots” for harm. Figure 2 presents a matrix plot of the distribution of the issues across the stages of care. The size of the boxes on the plot reflects the number of issues occurring at that stage, and a solid outline indicates that the number is more than would be expected based on the chi-square test. From this figure, it is clear that there are hot spots for harm in both the consultation and referral/follow-up stages, as well as when a complaint occurs across multiple stages.

### Blind spots

Three types of blind spots (i.e. areas of care that are difficult to observe by staff members or that are incorrectly observed) were identified.

1. *“Entry/Exit” blind spot*. Of the issues within the complaints presented in Table 2, almost one third (*n* = 131, 30%) occurred at the boundaries of care, that is, stages 1 (Accessing Care) and 4 (Referral/Follow-up). Issues included not being able to make an appointment with the GP, not being referred for specialist care, and not being scheduled for follow-up tests.
2. *“Errors of omission” blind spot*. Issues assigned to the “Quality,” “Communication,” and “Listening” categories were considered “Errors of omission,” based on their definition in the original HCAT.^2^ Therefore, over one third of issues in this sample were classified as being “Errors of omission” (*n* = 161, 37%). Examples of these issues included not listening to patients regarding allergies, failure to conduct a thorough examination, and not sending test results.
3. *“Systemic problems” blind spot.* Following the logistic regression, it was found that there was indeed a systemic problems blind spot. A systemic problems blind spot is difficult to observe by staff members, as only the patient is present for the series of issues that occur across different stages of their care pathway. Complaints with more issues were associated with an increased likelihood of harm, that is, for each additional issue within a complaint, the likelihood of harm to the patient increased (odds ratio = 2.02, CI = 1.27–3.23, *P* < 0.05). A total of 132 complaints (57%) contained more than 1 issue. The full model can be found in Supplementary Data 1.

## Discussion

The HCAT(GP) is a reliable framework that can support the systematic analysis of complaints. This study highlighted a number of hot spots and blind spots across general practice care in Ireland through the application of the HCAT(GP) to healthcare complaints.

The application of the HCAT(GP) enabled the systematic categorization of complaints made about general practice in Ireland, and allowed for further analysis of the trends across these complaints. Overall, issues emerged in each of the categories and domains of the HCAT(GP). In contrast to analyses of hospital complaints, where clinical issues dominated the findings,^2^ relationship issues (e.g. problems with listening, communication, and respect or patient rights) emerged most frequently from the GP complaints. This frequency of relationship issues within complaints indicated the potential importance of the doctor–patient relationship in general practice. This is arguably more important in primary care as patients have relationships with their GPs that last for many years.^18^ While there may have been limited harm associated with relationship issues, the frequency of this category indicates the need to explore this further. Communication skills training, an emphasis on patient-centered care, and rapport building could all be utilized to help address relationship issues faced by patients when receiving care.^19^

Clinical and management issues also were prevalent in the analysis, with 24% (*n* = 105) of issues pertaining to institutional processes, and 21% (*n* = 89) to problems with quality of care. Complaints often incorporated issues from across several categories. This phenomenon also occurred in hospital complaints.^2^ The wide scope of issues from this complaints analysis suggests that there is a need to take a broad overview of quality and safety improvement in general practice, and not focus solely on clinical issues. A previous systematic review highlighted the prevalence of clinical issues in general practice complaints.^20^ However, this study also underlined the need to address relationship and management issues. It is important these trends are explored further in other contexts to establish whether they persist.

Hot spots (areas of care where harm or near-misses occur frequently) and blind spots (areas of care that cannot be easily observed by staff) in care emerged from the analysis. A hot spot for harm was identified in the consultation stage. Patient safety research in secondary care is often centered on identifying error and clinical issues,^21^ however this is less of a focus in general practice research.^22^ The harm hot spot emerging at the consultation stage in this study indicates that errors do occur within the general practice context, reflecting recent research on the prevalence of patient safety issues within general practice.^23^ Another harm hot spot identified was at the “follow-up/referral” stage. Harm emerging at the boundaries of care is indicative of the recognized gap faced by patients when transitioning between different aspects of the health service.^24^ GPs tend to work independently, and lack the administrative and clinical support afforded to their hospital colleagues.^25^ In Ireland, 25% of GPs work in single-handed practices, which could contribute to issues at the boundaries of care, along with a perceived lack of support on referrals from hospital departments.^26,27^ Future research should further explore the complaints in order to understand the full extent of the phenomena occurring at these stages.^2^ Interventions could in turn be developed based on these findings to improve quality of care at these points of the care pathway. There is also a need to examine other types of hot spots that could occur in general practice, particularly “near-miss” hot spots. With a larger sample of complaints, “near-miss” hot spots where there are a lot of high-severity issues that don’t report harm, could be identified.^2^ Similarly, hot spots where catastrophic harm occurs frequently could be analyzed.

Several blind spots in general practice were identified in the analysis. These included the “entry/exit,” “systemic problems,” and “errors of omission” blind spots. The identification of a systemic problems blind spot highlighted that if a patient experienced poor care at multiple stages during their interaction with their general practice, they were more likely to report harm, something which is echoed in analyses of hospital complaints.^2^ It is vital to have oversight on these blind spots as patients are often the only people who witness their care across the entire system.^2^ This is an aspect of care that cannot be accessed using other forms of patient safety monitoring tools, and therefore highlights the potential for complaints to be used in tandem with these other tools (e.g. patient record review, global trigger tools, safety climate questionnaires^3,28,29^). Similarly, errors of omission are rarely identified through other means, as they are rarely observed, or when they are, responsibility is rarely taken.^30^ However, errors of omission are prevalent in healthcare, and these findings reiterate the importance of healthcare complaints for identifying errors of omission.^2^ Safety and quality improvement must incorporate the learning from blind spots to ensure issues are dealt with. Utilizing the data on blind spots that emerge from complaints could help identify issues as they emerge, and prevent harm from occurring to patients.^2^

### Implications for research and practice

Several important implications have emerged from this study. First, it has highlighted the difficulties experienced by patients at the transitional points of general practice. This emphasizes the importance of systematic and centralized analysis of complaints across an entire healthcare system. The HCAT(GP)^9^ could be used in conjunction with the HCAT for hospital complaints^10^ to give a broader overview of the transition from primary to secondary care, and identify which areas that require improvement. Complaints that incorporate elements from both primary and secondary care can now be analyzed comprehensively. Future research could ascertain the benefits of having a tool which can identify issues at these transitions of care in both hospital and GP settings.

Second, future research could explore collaborating with general practice stakeholders to consider interventions to address the key findings from the analysis, help prioritize areas for quality improvement, and identify ways through which interventions can be implemented. Having applied the HCAT(GP) to complaints in this study, hot spots (e.g. in the consultation stage) and blind spots in care (e.g. errors of omission) have been described. Translating these findings from research into practice will be vital, and this is often a difficult gap to bridge.^31^ Involving stakeholders and service users in prioritizing issues for quality improvement would help translate these findings into tangible improvements in practice.^32^ This study examined trends across an entire dataset of complaints. Using the stakeholders to prioritize specific issues and explore the complaints qualitatively would help identify improvement initiatives. For example, a subsample of just high-severity issues in complaints, or just catastrophic harm, could be examined in a stakeholder consultation and prioritized for quality improvement. This work, as well as the insight into the higher level issues across the system gained from this paper, would facilitate improvement initiatives which both tackle issues on the ground and transfer the learning to the wider system.^2,33^

### Limitations

This study had several limitations that should be noted. First, the sample size was limited. There is no single, centralized database of GP complaints in Ireland. As such, it is challenging to obtain a large sample of complaints. In addition, due to the requirement to redact the complaints, there was a large burden on the data controllers, limiting the feasibility of gathering a large number of complaints. As a result of this limited sample size, more detailed analyses on hot spots and blind spots could not be run. The small sample did not allow for the identification of a near-miss or catastrophic harm hot spots. A second limitation is that 2 databases were used. The first was the HSE database, referring only to public patients, and the second encompassed medical council complaints about GPs insured by 1 company. It is possible that complaints differed in characteristics across these 2 databases, and that the medical council complaints could have been of a higher severity. Future research should explore the differences between complaints received through different pathways. The next limitation is that the complaints were all collected prior to the beginning of the COVID-19 pandemic. Future research must determine whether the HCAT(GP) can reliably analyze and classify complaints in light of the changes to general practice that may result from a pandemic (e.g. increase in online consultations). Another limitation is the inherent issue with complaints research, that it is based solely on the perspective of the complainant. While this is typical within complaints research,^10^ it does mean that in some instances, complaints may not be accurate. Finally, limited reliability testing was conducted on the tool for this study. The overall reliability of the tool was calculated, however variation in reliability between different aspects of the tool was not assessed, as it is in other papers on the HCAT.^2,34^ However, the HCAT(GP) was assessed for reliability in previous work.^9^

## Conclusion

Healthcare complaints are a valuable, but underutilized, source of data to improve the quality and safety of general practice. This study has demonstrated the utility of the HCAT(GP) tool to support the systematic analysis of patient complaints about general practice, and has identified the need to focus on hot spots and blind spots across the care pathway. This information could be used to support an evidence-based approach to service improvement.

## Supplementary Material

cmab109_suppl_Supplementary_Data_1

cmab109_suppl_Supplementary_Material

## Data Availability

Data can be accessed upon request to the corresponding author.
