# Supplementary material for: Identifying hot spots for harm and blind spots across the care pathway from patient complaints about general practice
Source: Fam Pract. 2021 Sep 19;39(4):579–85. doi: 10.1093/fampra/cmab109 (PMC9295605; doi:10.1093/fampra/cmab109)
Supplement: cmab109_suppl_Supplementary_Data_1 [file cmab109_suppl_supplementary_data_1.docx]

**Supplementary Data 1. Full logistic regression model for likelihood of harm occurring.**

Harm is more likely to occur as the number of issues within a complaint increases.

The intercept refers to no harm.

Harm ~ No of issues + No of stages

| **Coefficients** | **Estimate** | **Std Error** | **Z value** | **p** |
| --- | --- | --- | --- | --- |
| Intercept | -1.286 | 0.356 | -3.614 | <.005* |
| Number of issues | 0.705 | 0.238 | 2.963 | <.005* |
| Number of stages | -0.005 | 0.320 | -0.016 | 0.987 |

*Significant at a p<.005 level
